# Trends in Artificial Intelligence Usage in Orthopaedic Surgery Residency Applications

**DOI:** 10.2106/JBJS.OA.25.00145

**Published:** 2025-11-19

**Authors:** Benjamin C. Hawthorne, Lisa M. Tamburini, Marissa A. Gedman, Rohan R. Patel, Tomer Korabelnikov, Michelle Ambrosio, Ian J. Wellington, Matthew E. Shuman, Scott Mallozzi, Hardeep Singh

**Affiliations:** 1 Department of Orthopaedic Surgery, University of Connecticut, Farmington, Connecticut

## Abstract

**Background:**

The public adoption of artificial intelligence (AI) tools such as ChatGPT has expanded rapidly in recent years, including growing use among healthcare professionals. The purpose of this study was to assess the use of AI and plagiarism in orthopaedic surgery residency applications across the 2022 to 2023, 2023 to 2024, and 2024 to 2025 application cycles.

**Methods:**

Deidentified letters of recommendation (LORs) and personal statements (PSs) from interviewed applicants across 3 consecutive application cycles at a single institution were analyzed. Blinded reviewers input the documents into an online platform designed to detect AI-generated language and plagiarism.

**Result:**

There was a statistically significant increase in the AI usage in LORs during the 2024 to 2025 application cycle (17.8%) compared with the 2023 to 2024 (5.0%) and 2022 to 2023 (5.6%) cycles (p < 0.001). Correspondingly, the originality scores of LORs significantly decreased in 2024 to 2025 (92.4%) compared with 2023 to 2024 (97.3%) and 2022 to 2023 (97.7%) (p < 0.001). By contrast, the AI usage in personal statements significantly decreased in 2024 to 2025 (43.5%) compared with 2023 to 2024 (60.3%) and 2022 to 2023 (65.2%) (p = 0.031). There was no significant difference in plagiarism scores across the 3 cycles for either LORs (p = 0.28) or PSs (p = 0.39).

**Conclusions:**

The 2024 to 2025 application cycle showed a marked increase in AI usage in letters of recommendation, while the usage of AI in personal statements declined. These trends reflect evolving patterns in how applicants and letter writers are integrating AI tools into the application process.

**Level of Evidence:**

Level III; Retrospective Cohort Study. See Instructions for Authors for a complete description of levels of evidence.

## Introduction

Over the past 3 years, artificial intelligence (AI) technologies have rapidly evolved and are more accessible to the public. This trend was catalyzed by the release of ChatGPT by OpenAI in November 2022. ChatGPT employs a subset of AI known as generative AI, which uses natural language processing and advanced machine learning algorithms to generate text that closely mimics human writing. Following ChatGPT's introduction, other generative AI models such as Microsoft Copilot and Google Bard were released in March 2023, further expanding access to these tools.

These models have been readily adopted by users, particularly in academic environments. In Fall 2022, shortly after the release of ChatGPT, an informal study found that 17% of Stanford students used the technology for assignments and examinations^1^. Despite early evidence of its integration in academic settings, limited data exist on how AI tools are being used by medical students applying for competitive residency programs.

Residency selection committees consider a wide range of factors during the evaluation process, including United States Medical Licensing Examination scores, clerkship performance, personal statements (PSs), and letters of recommendation (LORs). The PS allows applicants to highlight relevant experiences and qualifications that make them a strong fit for a residency program. Similarly, the LOR provides faculty an opportunity to promote applicant characteristics and provide a personal evaluation for their colleagues in the field. Given their narrative nature, the PS and LOR are particularly amendable to AI-assisted writing.

There are potential advantages to the use of AI in writing, including improved efficiency, assistance in brainstorming, and the reduction of biased language^2,3^. However, concerns remain regarding the authenticity and originality of AI-generated content, as it may lack the personal connection and emotional intelligence inherent in human-written prose.^4-6^

The purpose of this study was to assess the use of AI and plagiarism in orthopaedic surgery residency applications across the 2022 to 2023, 2023 to 2024, and 2024 to 2025 application cycles. We hypothesized that the prevalence of AI-generated content in LORs and PSs would increase over time, reflecting broader trends in AI adoption across academic and professional settings.

## Materials and Methods

This study analyzed LORs and PSs submitted by fourth-year medical students applying to the University of Connecticut Orthopaedic Surgery Residency program across the 2022 to 2023, 2023 to 2024, and 2024 to 2025 application cycles. Before data collection, Institutional Review Board approval was obtained (Protocol #25X-158-2).

An individual blinded to the study deidentified all LORs and PSs and assigned a unique code to ensure applicant confidentiality while enabling accurate data tracking. Owing to the large number of applications received annually (>700), the study was limited to documents submitted by applicants who were invited for interviews.

AI usage and originality were assessed using Orginality.ai, a commercially available AI detection tool previously validated in academic research. A study by Walters demonstrated that Originality.ai accurately identified 100% of documents generated by GPT-4 and 95% of human-written documents, with an overall accuracy rate of 98%^7^. The platform is trained on the top 35 generative AI large language models.

Two blinded reviewers independently uploaded the deidentified documents into the AI detection software. The platform produced a likelihood of AI usage as an originality score for each document. This score represented the probability of human-generated vs AI-generated language. For analysis purposes, documents with a 100% likelihood of AI-generated language were assigned a score of 0, and those with a 100% likelihood of being human composed were assigned a score of 100. Plagiarism was calculated by dividing the number of plagiarized words by the total word count of the document.

LOR and PS with an originality score less than 100 were classified as likely to have been generated or influenced by AI. For each application cycle, the average likelihood of AI usage and average originality score were calculated for both LORs and PSs. Plagiarism was also assessed. The number of documents with any level of plagiarism was recorded, and average plagiarism scores were calculated for each cohort.

Kruskal-Wallis tests were used to compare the differences in originality scores for use of AI and plagiarism between the 3 cohorts. When significant differences were found, post hoc pairwise comparisons were conducted using Mann-Whitney tests.

## Results

A total of 231 LORs and 66 personal statements were analyzed from 66 interviewed applicants in the 2022 to 2023 application cycle. In 2023 to 2024, 179 LORs and 58 personal statements were reviewed from 58 interviewed applicants. From the 2024 to 2025 cycle, 250 LORs and 69 personal statements were reviewed from 69 interviewed applicants.

**Fig. 1:**
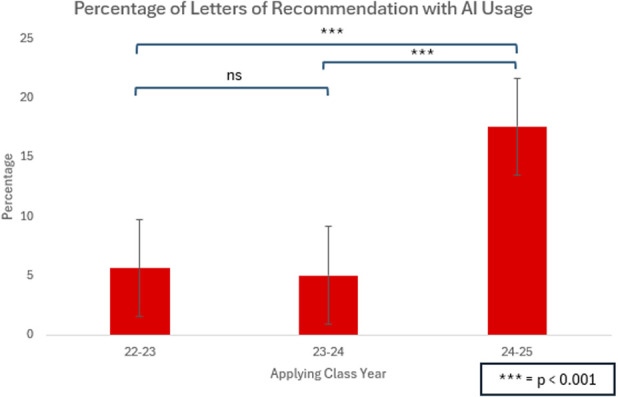
There was a significant increase in AI usage in letters of recommendation of the 2024 to 2025 class (17.8%) compared with the 2022 to 2023 (5.6%) and 2023 to 2024 (5.0%) classes (p < 0.001). AI = artificial intelligence.

**Fig. 2:**
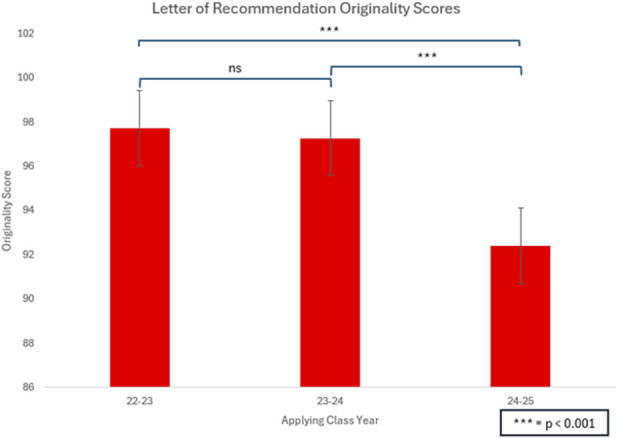
There was a significant decrease in the originality score of letters of recommendation of the 2024 to 2025 class (92.4) compared with the 2022 to 2023 (97.7) and 2023 to 2024 (97.3) classes (p < 0.001).

Among LORs from 2022 to 2023 class, 13 of 231 (5.6%) were found to have some likelihood of AI involvement. A similar proportion was observed in the 2023 to 2024 class, with 9 of 179 LORs (5.0%) identified as AI-assisted, and an average originality score of 97.29 (p = 0.82; compared with 2022-2023). By contrast, the 2024 to 2025 cycle demonstrated a significant increase in AI usage in 44 of 250 LORs (17.6%), p < 0.001 (Fig. 1). The average originality score for the 2024 to 2025 LORs was 92.4% which was significantly lower than the originality score of the 2022 to 2023 (97.7%) and 2023 to 2024 (97.3%) cohorts (p < 0.001) (Fig. 2).

Plagiarism in LORs was minimal across all 3 cohorts. Two LORs from 2022 to 2023 cycle contained plagiarized contents (5% and 10%), while 2 LORs from the 2023 to 2024 contained 4% and 6% plagiarized content, respectively. No LORs from the 2024 to 2025 cycles showed evidence of plagiarism. There was no statistically significant difference in LOR plagiarism scores among the cohorts (p = 0.28).

**Fig. 3:**
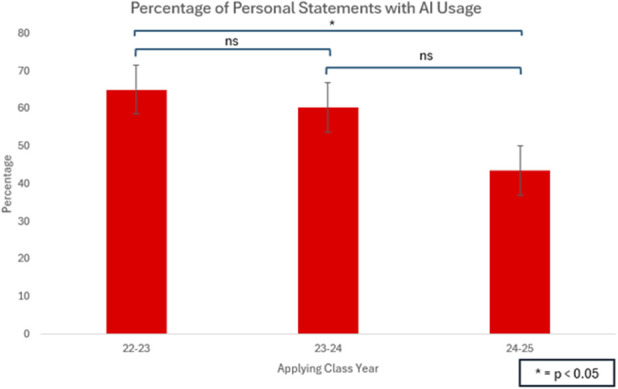
There was a significant decrease in AI usage in personal statements of the 2024 to 2025 class (43.5%) compared with the 2022 to 2023 (65.2%) and 2023 to 2024 (60.3%) classes (p = 0.033). AI = artificial intelligence.

In contrast to the trend observed in LORs, AI usage in PSs decreased over time. For the 2022 to 2023 cohort, 43 of 66 (65.2%) personal statements were likely influenced by AI. This percentage dropped slightly in the 2023 to 2024 cohort to 60.3% (35 of 58) and significantly decreased to 43.5% (30 of 69) in the 2024 to 2025 cohort (p = 0.03) (Fig. 3).

Average originality scores for PSs did not differ significantly between cohort, with 77.3% in 2022 to 2023, 72.7% in 2023 to 2024, and 76.1% in 2024 to 2025 (p = 0.23). Similarly, plagiarism scores in PSs showed no significant difference across the 3 cohorts (p = 0.39).

## Discussion

This study observed a marked increase in AI usage within LORs across 3 consecutive orthopaedic surgery residency application cycles, rising from 5.6% in 2022 to 2023 to 17.6% in 2024 to 2025. However, the study observed a significant decrease in AI usage in personal statement across the past 3 application cycles.

The growing accessibility of AI platforms presents a new challenge for residency selection committees, particularly in assessing the authenticity of narrative components such as letters of recommendations and personal statements. These sections of the applications, due to their open-ended and narrative format, are amendable to AI-assisted writing.

Although the use of AI for LORs may improve consistency and efficiency, it raises concerns about bias, loss of individuality, and a potential decrease in the evaluative value of the letter. As the use of AI in generating LORs becomes more widespread, it will likely be increasingly difficult for committees to assess applicants based on personalized insights and qualitative judgements. Standardization through AI could mask an applicant’s unique attributes or inflate perceived strengths, potentially undermining the purpose of these letters.^8^

Personal statements are intended to allow applicants to express their unique motivations, experiences, and value. These elements are best conveyed through introspection and authenticity. Although multiple studies have shown individuals are generally poor at distinguishing between human-generated or AI-generated text, evidence shows that selection committee members tend to favor human-authored personal statements when evaluating motivations, interest, and career goals.^9-13^

Although generative AI can be used to write entire personal statements, it can also be used to revise what has been written to improve readability and to help brainstorm ideas^14^. For instance, prior research has described how applicants can use tools such as ChatGPT to either generate full drafts or enhance prewritten statements^15^. These tools may be used responsibly to polish content, but their misuse can lead to statements that lack personal authenticity and emotional resonance.

Interestingly, our study found an overall decrease in the likelihood of AI usage in personal statements from 2022 to 2023 cycle (65.2%) to 2024 to 2025 cycle (43.5%). This trend could reflect evolving perceptions of AI in academic and professional writing. Applicants may be increasingly aware of the ethical concerns or wary of the impersonal tone associated with AI-generated prose. Alternatively, applicants may have found better use of AI in helping to reword and edit personal statements to allow personal experiences to be shared in a refined way. In addition, the Electronic Residency Application Service (ERAS) has introduced a certification requirement in which applicants must affirm that their personal statement is not the product of artificial intelligence^16^. These factors may have contributed to the observed decline in AI usage in personal statements. However, despite firm statements from the AAMC that personal statements should not be the product of AI, it is concerning that this study found that more than 40% of students appear to still be using AI to write their personal statements.

As AI tools continue to evolve and integrate into daily workflows, residency programs must consider how to address their influence in the application process. The findings suggest that AI usage patterns differ between application components and have shifted over time. Although LORs appear to be increasingly AI-assisted, applicants may be exercising more discretion in their use of AI for personal statements. Residency selection committees should be mindful of these trends and may wish to develop thoughtful guidelines regarding the appropriate and ethical use of AI in residency applications.

## Limitations

This study is not without its limitations. First, the study was performed at a single institution which may limit the generalizability of the results to other orthopaedic surgery residency programs. Second, a single, verified AI detector was used for this study and different results may have been found had we used other models. Third, owing to concerns of linking analysis of applications to applicants, there were limited demographic data for the study to analyze which individuals are using AI more. Finally, this study did not analyze how AI usage in applications affected the credibility of the application as perceived by application reviewers, given that the goal was to observe overall trends in the usage of AI in the application process.

## Conclusions

The 2024 to 2025 application cycle showed a marked increase in AI usage in letters of recommendation, while the usage of AI in personal statements declined. These trends reflect evolving patterns in how applicants and letter writers are integrating AI tools into the application process.
